# IFI16 induces inflammation in hepatitis B virus-associated glomerulonephritis by regulating the Caspase-1/ IL-1 ß pathway

**DOI:** 10.1186/s13000-022-01220-9

**Published:** 2022-04-23

**Authors:** Li Liu, Shuangshuang Xie, Cheng Li, Yue Guo, Xiaoyan Liu, Xiuhua Zhao, Qiang Li, Wenjun Du

**Affiliations:** Department of Liver Diseases, Shandong Public Health Clinical Center, Shandong University, Jinan, 250000 China

**Keywords:** IFI16, HBV-DNA, HBV-GN, Caspase-1, IL-1β

## Abstract

**Aims and background:**

IFI16 plays an important role in innate immunity against invasive microbial infection by sensing double-stranded DNA viruses due to caspase-1-dependent inflammasome activation and subsequent maturation and secretion of IL-1β. However, the role of IFI16 in regulating the immune response to viruses in Hepatitis B Virus-Associated Glomerulonephritis (HBV-GN), especially in sensing hepatitis B virus (HBV), has not been determined. In this study, we investigated the inflammatory role of IFI16 in HBV-GN.

**Methods:**

A total 75 kidney tissue including 50 HBV-GN and 25 chronic glomerulonephritis (CCN) were collected to determine the expression of IFI16, Caspase-1 and IL-1β using immunohistochemistry (IHC), then the correlation between them was analyzed. In vitro, the primary human glomerular mesangial (HGM) cells and HEK-293 T cell lines were used in this study. The cell lines were both co-transfected with HBVDNA and overexpression or silencing IFI16. Quantitative Real-time PCR and western blotting were used to determine the expression of IFI16, Caspase-1 and IL-1β.

**Results:**

IFI16 expression in HBV-GN biopsies (80.0%) was significantly higher than in CGN (24.0%) and positively correlated with HBVDNA,caspase-1 and IL-1β expression in HBV-GN. Meanwhile, over expression of IFI16 increased caspase-1 and IL-1β expression in HBV-infected HGM and HEK-293 T cell lines, knockdown of IFI16 mRNA by siRNA resulted in downregulation of the caspase-1 and IL-1β expression in both cell lines.

**Conclusions:**

The elevation of IFI16 during HBV infection or replication may contribute to renal damage due to inflammation, thus providing a putative therapeutic target and a new avenue for researching the pathogenesis of HBV-GN.

## Introduction

Hepatitis B virus (HBV) infection is an important public health problem worldwide, which causes both hepatic and extrahepatic organ injures. Hepatitis B virus-associated glomerulonephritis (HBV-GN) is the most common and well described extrahepatic manifestations of chronic HBV infection [1]. HBV-GN was first reported by Combes et al. in 1971 [2], and since then more cases have been described all over the world. The most widely accepted mechanism is the deposition of immune complexes of viral antigen and host antibody in renal lesions by persistent HBV infection [3]. However, the immune pathogenesis of HBV-GN remains unclear.

IFI16 (Interferon-γ-inducible protein 16) belongs to Pyrin-Hin200(HIN-200) family, which plays an important role in antiviral and immunomodulatory activities [4, 5]. As innate immune sensors, IFI16 recognizes both cytosolic and nuclear double-stranded DNA (dsDNA) from invasion of many viruses, such as vaccinia virus (VACV), herpes simplex virus 1 (HSV-1), and Kaposi sarcoma-associated herpesvirus (KSHV) [6–8]. Following the detection of dsDNA viruses, IFI16 combines with apoptosis-associated speck-like protein (ASC) and triggers downstream stimulator of interferon genes-TANK-binding kinase1-interferon regulatory factor 3 (STING-TBK1-IRF3) signaling pathway [8, 9]. This response leads to the activation of caspase-1, which cleaves proinflammatory type I interferon (IFN-I) and interleukin-1ß (IL-1ß) to generate their active forms [10]. These inflammatory cytokines play critical roles in the host immunity against viral infection. In addition, overexpression of IFI16 leads to autoimmune diseases such as systemic lupus erythematosus (SLE) and Sjogren Syndrome (SjS) in which DNA is a major autoimmune target [11].

Recent evidences show that IFI16 expression is associated with the degree of inflammation in acute and chronic hepatitis B [12, 13]. However, little is known about the roles of DNA sensor IFI16 in HBV-GN pathogenesis. Previous studies showed that HBV DNA was also identified in the cells of the nephron unit and interstitial tissue from HBV-GN patients [14]. We propose that cytoplasmic and nuclear HBV-DNA in the renal tissues has high possibilities to be recognized by IFI16. The potential binding of IFI16 to HBV-DNA may lead to the activation of caspase-1 and subsequent maturation and secretion of IL-1β. This cascade of events leads to the development of the inflammasome, which may be responsible for the renal damage seen in HBV-GN patients.

To determine whether IFI16 expression is associated with HBV infection, we compared the expression of IFI16 in HBV-GN and chronic glomerulonephritis (CGN). Here we show for the first time that higher IFI16 levels in HBV-GN compared with CGN, and IFI16 expression levels are associated with HBV-GN inflammation degree. Further we explored the relationship between the expression of IFI16, caspase-1 and IL-1β in HBV-GN group. Results showed that caspase-1 and IL-1β expression in HBV-GN was positively correlated with the expression of IFI16.

To further identify the correlation between IFI16 expression and inflammation in HBV-GN, we performed in vitro experiments using primary human glomerular mesangial (HGM) cells and HEK-293 T cells. The cell lines were co-transfected with HBVDNA and overexpression or silencing IFI16. Then the expression levels of IFI16、caspase-1 and IL-1β were investigated. We demonstrated the overexpression of IFI16 increased expression of caspase-1 and IL-1β, while knockdown of IFI16 resulted in the efficient reduction of those inflammatory cytokines expression in the cells transfected with HBV. Thus, IFI16 may play an important role in the development and progression of HBV-GN inflammation.

## Materials and methods

### Patients

Our retrospective study was approved by the ethics committee of Jinan Infectious Disease Hospital (JCLL-2016-04). A total of 75 patients diagnosed with chronic nephritis, identified between 2008 and 2016 at Jinan Infectious Disease Hospital and Qilu Hospital of Shandong University Shandong, China, were included in the study. The experimental group consisted of 50 HBV-GN patients, the negative control group consisted of 25 CGN patients, CGN should be ruled out the following criteria:1) PCR detection of HBV-DNA and Elisa detection of HBV-associated antigen including HBsAg and HBeAg to test HBV infection; 2) PCR detection of EBV-DNA, HSV-DNA, HCVM-DNA and HPV-DNA to test the common ds-DNA virus infection; 3) with auto-immune disease. Each patient received kidney puncture biopsy under ultrasound guidance to attain renal tissue for diagnosis and subsequent research. Participation was dependent upon fulfillment of the following criteria:(1) patients must not use an immune agent or antiviral agent in the past 3 months; (2) patients must not have HAV, HCV, HDV, HEV or HIV co-infection; (3) patients must not have a history or current evidence of secondary glomerulonephritis; and (4) consent for participation must have been obtained from those who participated. The basic characteristics of patients are listed in Table 1.

**Table 1:** The basic characteristics of the populations enrolled in the study

GROUP	HBV-GN	CCN
case	50	25
sex(M/F)	35/15	15/10
age (years)	37 (18–65)	41 (19–61)
BUN (mmol/L)	9.28 (7.51–22.64)	10.27 (8.72–25.51)
Cr (μmol/L)	176 (138–227)	184 (133–248)
Log10 (HBV DNA)	5.42 (0–8.54)	0
HBeAg-positive	35 (70%)	0

Data are shown as median and range

*HBV-GN* Hepatitis B virus associated glomerulonephritis, *CCN* chronic glomerulonephritis, *HBeAg* hepatitis B e antigen

### Diagnosis of HBV-GN, CGN and pathological classification of HBV-GN

**Fig. 1:**
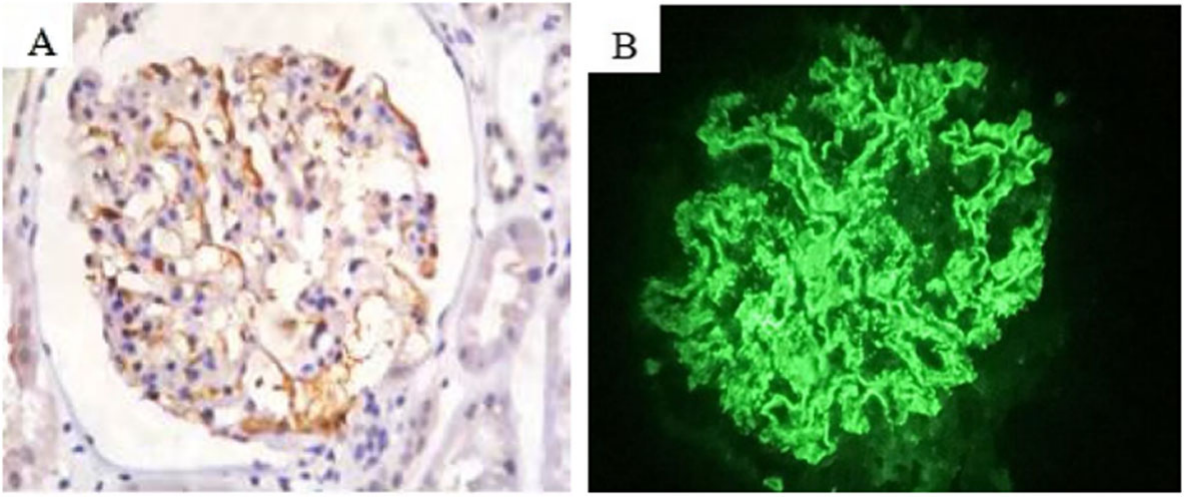
**A** The immunohistochemical staining of HBcAg was positive, and the brown-yellow granules were distributed along the capillary wall of the glomerulus. IHC, 400×. **B** IgG deposits along the glomerular capillary wall and mesangial area, Immunofluorescence, 400×

The diagnostic criteria used for CGN and HBV-GN were in accordance with the 2002 Kidney Disease Outcome Quality Initiative (K/DOQI), edited by the National Kidney Foundation (NKF) [15]. The diagnosis of HBV-GN was confirmed by pathology. The pathological classification of and diagnostic criteria used for HBV-GN were in accordance with 1990 WHO classification criteria [16]. Frozen slices from biopsies of the 50 HBV-GN patients were kept in a low-temperature freezer. Monoclonal goat-anti-human HBsAg and HBcAg antibodies were purchased from Dako (Denmark), antibodies against IgA, IgG, IgM, C3 and C1q complement component. Fluorescently-labeled IgA, IgG, IgM, C3 and C1q rabbit-anti-human antibodies were purchased from Dako. Immunohistochemical staining for HBsAg and HBcAg in renal biopsies was used or electron microscope detection for HBV to confirm the diagnosis (Fig. 1). For HBV-GN patients with undetectable HBsAg or HBcAg in renal tissue, HBV was detected using the JCM-6000 scanning electron microscope from Jeol. Ltd. (Japan). Sections from all biopsy specimens were stained routinely with hematoxylin and eosin (H&E), periodic acid-sliver methenamine (PASM), Masson’s trichrome.

### Immunohistochemistry and scoring

Immunohistochemistry was carried out using standard techniques. Renal tissue specimens were first fixed in 10% formalin, then the tissue was cut, dehydrated, dipped in wax, embedded and sectioned. These sections were then placed on slides, baked, placed into xylene, cleared of the wax, rehydrated using graded ethanol and immersed in 0.3% hydrogen peroxide for 5 min to reduce non-specific background staining caused by endogenous peroxidase. The slides were then washed with PBS buffer three times for 5 min each, placed in citrate buffer solution at a pH of 6.0 and then into a high temperature pressure pot to recover the tissue antigen. After being heated, the slides were cooled and restored at room temperature, washed three more times in PBS buffer and incubated with IFI16 (ab55328, rabbit anti-human polyclonal antibody; Abcam, USA), caspase-1 (sc-56,063, mouse anti-human polyclonal anti- body; Santa Cruz Biotechnology Inc., USA) and IL-1β antibodies (ab2105, rabbit anti-human polyclonal anti-body; Abcam), respectively. The slides were then placed in a 4 °C refrigerator overnight. The next day, the slides were washed with PBS buffer three times, each time lasting longer than 5 minu, then incubated with the secondary antibody PV-9000 (universal antibody) at 37 °C for 10 min, washed with PBS buffer, and DAB staining was applied. The stain was terminated using running water, then the slides were washed with hydrochloric acid alcohol for differentiation. Lastly, the slides were washed with distilled water, cleared with xylene and mounted. Appearance of a tan stain in the cytoplasm signaled positive expression of the protein. After staining, scores were assigned based on stain intensity and percentage of positive cells as follows: For stain intensity, a score of 0 was given for no brown staining (i.e., no cells stained), 1 for light brown, 2 for brown and 3 for dark brown; for percentage of positive cells, a score of 0 was given for fewer than 5% positive cells, 1 for 5 to 30%, 2 for 30 to 60% and 3 for greater than 60%. Scores for stain intensity and percent positive were then added together, and a negative sign (−) was assigned for scores totaling 0, mildly positive (+) for scores between 1 and 3, moderately positive (++) for scores between 4 and 6 and strongly positive (+++) for scores greater than 7.

### Cell lines and reagents

The human glomerular mesangial (HGM) cell line and HEK-293 T cell line used in this study were purchased from the cell bank of the Chinese academy of sciences (Shanghai, China) and cultured in Dulbecco’s modified Eagle’s medium (DMEM) supplemented with 10% FBS (Life Technologies, Carlsbad, CA, USA), ampicillin and streptomycin at 37 °C, 5% CO_2_ conditions. HBV expression plasmids were constructed with a pcDNA3.0 vector. The 1.1-fold over length HBV genome was cloned into the pcDNA3 vector to generate pcDNA3.0–1.1HBVDNA, 1.1HBV as the expression gene and ampicillin resistance for antibiotic selection (Amresco, Penn- sylvania, USA). We designed three kinds of IFI16-siRNAs for this experiment, which were synthesized by Gene Pharma (Shanghai, China). the siRNA was synthesized accordingly:

siRNA #1: GGAAGUGGAUGCUACUUCAdTdT;

siRNA #2: GGAAUAUGAUAGUCUCCUAdTdT;

siRNA #3: GGAAGUGGAUGCUACUUCAdTdT;

siRNA negative control: GAUGAGAUUAGAUACUCUCdTdT.

IFI16-siRNA#2 was selected as the most effective silencer compared with the others, which was used for the following experiment (Fig. 4A). IFI16, Caspase-1 and IL- 1β antibodies were obtained from Cell Signaling Tech Abcam (Cambridge, MA, USA).

### Cell transfection

HGM cell line and HEK-293 T cell line were seeded into 12-well plates, then the cells were transfected with either pcDNA3.0–1.1HBVDNA- IFI16 and negative control for overexpression studies, or with siIFI16 and a scrambled siRNA for knockdown studies. Lipofectamine 2000 (Invitrogen) was used according to the manufacturer’s instructions with minor modifications for transfection studies. IFI16 overexpression and knockdown were confirmed by qRT-PCR and Western blot 48 h post transfection.

### Western blots

According to the manufacturer’s instructions, the whole cell protein extracts were prepared and were separated using 10% sodium dodecyl sulphate polyacrylamide gel electrophoresis. Proteins were then transferred to a polyvinylidene difluoride membrane (Millipore, Bed- ford, MA, USA), according to the instruction manual. Filters were blocked overnight in 5% w/v low- fat dry milk in 10 mmol/L Tris- HCl, pH 7.5, 0.1 mol/L NaCl and 0.1% Tween- 20 and incubated with primary antibodies overnight at 4 °C. After washing with TBST buffer, the blots were then incubated with HRP- conjugated secondary antibody for 2 h at room temperature. After washing with TBST buffer, Immunoreactive bands were visualized using the ECL-Plus reagent (Millipore, Billerica, MA, USA). GAPDH was used as the loading control in the Western blotting.

### RNA isolation and qRT-PCR

Total RNA was isolated using Trizol RNA reagent (Invitrogen, California, USA). Quantitative real- time PCR was performed, and the expression levels of IFI16, caspase-1 and Il-1ß mRNA were normalized to GAPDH for gene expression. The primers are listed in Table 2.

**Table 2:** Primers’ sequences used in qRT-PCR in this study

ID	Sequence (5′-3′)
IFI16 F	AGCTCAGAACCCGAAAACAG
IFI16 R	TCTGTGTAGCCACTGTAGCA
caspase-1 F	GTTCCATGGGTGAAGGTACA
caspase-1 R	GACATTCCCTTCTGAGCCTG
Il-1ß F	AGCTACGAATCTCCGACCAC
Il-1ß R	CGTTATCCCATGTGTCGAAGAA
GAPDH F	TGTTCGTCATGGGTGTGAAC
GAPDH R	ATGGCATGGACTGTGGTCAT

*F* forward primer, *R* reversed primer

### Statistical analysis

The SPSS program (version 19.0) and GraphPad Prism (version 8.0) were used for analysis. Measurement data was described as mean ± standard deviation. Background factors were compared using Student’s-test (numerical data) or the Chisquare test (categorical data). Spearman’s two-tailed test was used for correlation analysis, and differences were regarded as significant if the *p* value was less than 0.05 on either side.

## Results

### Expression of IFI16 was significantly high in HBV-GN compared with CGN

**Fig. 2:**
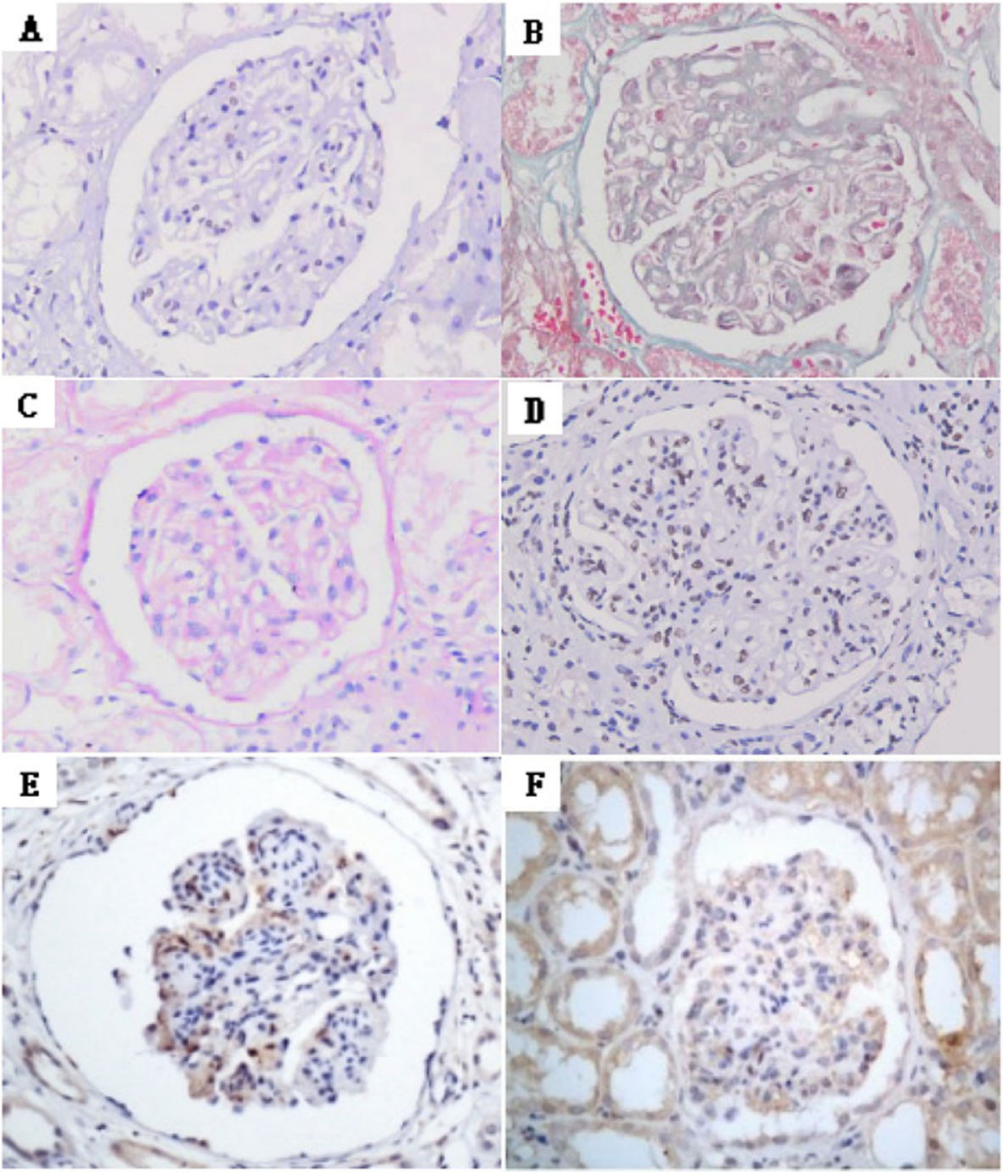
**A** Light microscopic manifestation of HBV-GN, HE,400×. **B** Light microscopic manifestation of HBV-GN, Masson,400×. **C** Light microscopic manifestation of HBV-GN,PAS, 400×. **D** IFI16 positive staining in nucleus of endothelial cells, mesangial cells and podocytes in HBV-GN, IHC 400×. **E** Caspase-1 positive staining in cytoplasmic and nucleus of glomerular endothelial cells and mesangial cells in HBV-GN,IHC 400×. **F** IL-1ß positive staining in cytoplasmic and nucleus of glomerular endothelial cells and mesangial cells in HBV-GN,IHC 400×

To measure the expression of IFI16 in the kidney of 50 HBV-GN and 25 CGN patients, we used immunohistochemistry with anti-IFI16 to probe sections of paraffin-embedded samples. IFI16 expression was observed in the nucleus, especially in the HBV-GN patients (Fig. 2D). Statistical analysis revealed that the positive expression rate of IFI16 in HBV-GN patients was significantly higher than in CGN patients (80% versus 24%, *p*<0.01) (Table 3).

**Table 3:** Expression of IFI16 was significantly high in HBV-GN compared with CGN

group	Tissue	Cases(n)	Age	Gender (M %)	IFI16				Positive Rate(%)
					–	+	++	+++	
HBV-GN	K	50	36.1 ± 12.7	35 (70%)	10	14	26	0	80
CGN	K	25	38.2 ± 15.5*	18 (72%)**	19	6	0	0	24***

*HBV-GN* Hepatitis B viral associated glomerulonephritis, *CGN* Chronic glomerulonephritis, *K* kidney, *M* Male. *compared with HBV-GN, (t = −0.072, *p* = 0.943); **compared with HBV-GN, (×2 = 0.032, *p* = 0.85); ***compared with HBV-GN, N, (× 2 = 30.091, *p* < 0.01)

### Expression of IFI16 was not correlated HBV parameters in HBV-GN

To further clarify the potential correlated factor associated with expression of IFI16, we analyzed the correlation with status of HBV-associated antigen deposited in kidney and pathological type of HBV-GN. the results indicated that there was no significant difference of IFI16 expression in sub-types of HBV-GN (*p* = 0.940) (Table 4). Then we tested for the relationship between IFI16 expression levels and HBV parameters. There was no correlation between IFI16 expression and HBVDNA levels(*p* = 0.247). also there was no difference of IFI16 expression levels between the HBeAg(+) and HBeAg(−) groups (*P* = 0.614) (Table 5).

**Table 4:** Expression of IFI16 was no correlation between pathological types of HBV-GN

		IFI16				
Pathological type	n(%)	–	+	++	X^2^	p
MsPGN	15 (30.0)	3	4	8		
MPGN	8 (16.0)	1	2	5		
MN	23 (46.0)	6	6	11		
MCG	4 (8.0)	0	2	2		
Total	50	10	14	26	2.875	0.850

*MsPGN* Mesangioproliferative glomerulonephritis, *MPGN* Membranoproliferative glomerulonephritis, *MN* Membranous nephropathy, *MCG* Minimal change glomerulopathy

**Table 5:** Expression of IFI16 and HBV parameters in HBV-GN

	n(%)	IFI16			X^2^	p
		–	+	++		
Gender, M	35 (70%)				0.131	0.937
Age (y)					2.598	0.627
≤ 20	5 (10%)	2 (4%)	2 (4%)	1 (2%)		
21–40	25 (50%)	4 (8%)	16 (32%)	5 (10%)		
≥ 41	20 (40%)	4 (8%)	8 (12%)	8 (12%)		
e-Ag (+)	35 (70%)	6 (12%)	18 (36%)	11 (22%)	0.975	0.614
HBV-DNA						
≥ 10^5^ cp/ml	16 (32%)	5 (10%)	5 (10%)	6 (12%)	6.097	0.247
< 10^5^cp/ml	34 (68%)	9 (18%)	11 (22%)	14 (28%)	6.573	0.256

M male; e-Ag, HBeAg; cp/ml, copies/ml

### Expression of IFI16 was positively correlated with Caspase-1 and IL-1ß in HBV-GN tissue

We investigated the expression of Caspase-1、IL-1ß in biopsied kidney tissue from 50 HBV-GN, The results showed the positive sites of caspase-1 and IL-1β were mainly located in glomeruli, with focal distribution in renal tubules and renal interstitium in the HBV-GN tissue. The correlation between expression of IFI16 and Caspase-1、IL-1ß was analyzed. Statistical analysis revealed that the expression of IFI16 was positively correlated with that of Caspase-1(r = 0.998, *p*<0.01) and IL-1ß (r = 0.953, *p*<0.05) (Table 6). These results suggested that the expression of IFI16 was correlated with that of inflammatory cytokine in HBV-GN tissue and elevation of IFI16 may responsible for inflammatory damage of HBV-GN.

**Table 6:** Expression of IFI16 was positively correlated with Caspase-1 and IL-1ß in HBV-GN tissue

IFI16	Caspase-1				Il-1ß			
	–	+	++	+++	–	+	++	+++
**+++**	0	0	0	0	0	0	0	0
**++**	5	5	14	2	3	10	12	1
**+**	0	4	10	0	1	4	9	0
**–**	6	4	0	0	5	4	1	0
rs	0.998				0.953			
p	< 0.01				< 0.05			

### Over expression of IFI16 promoted inflammation in vitro

**Fig. 3:**
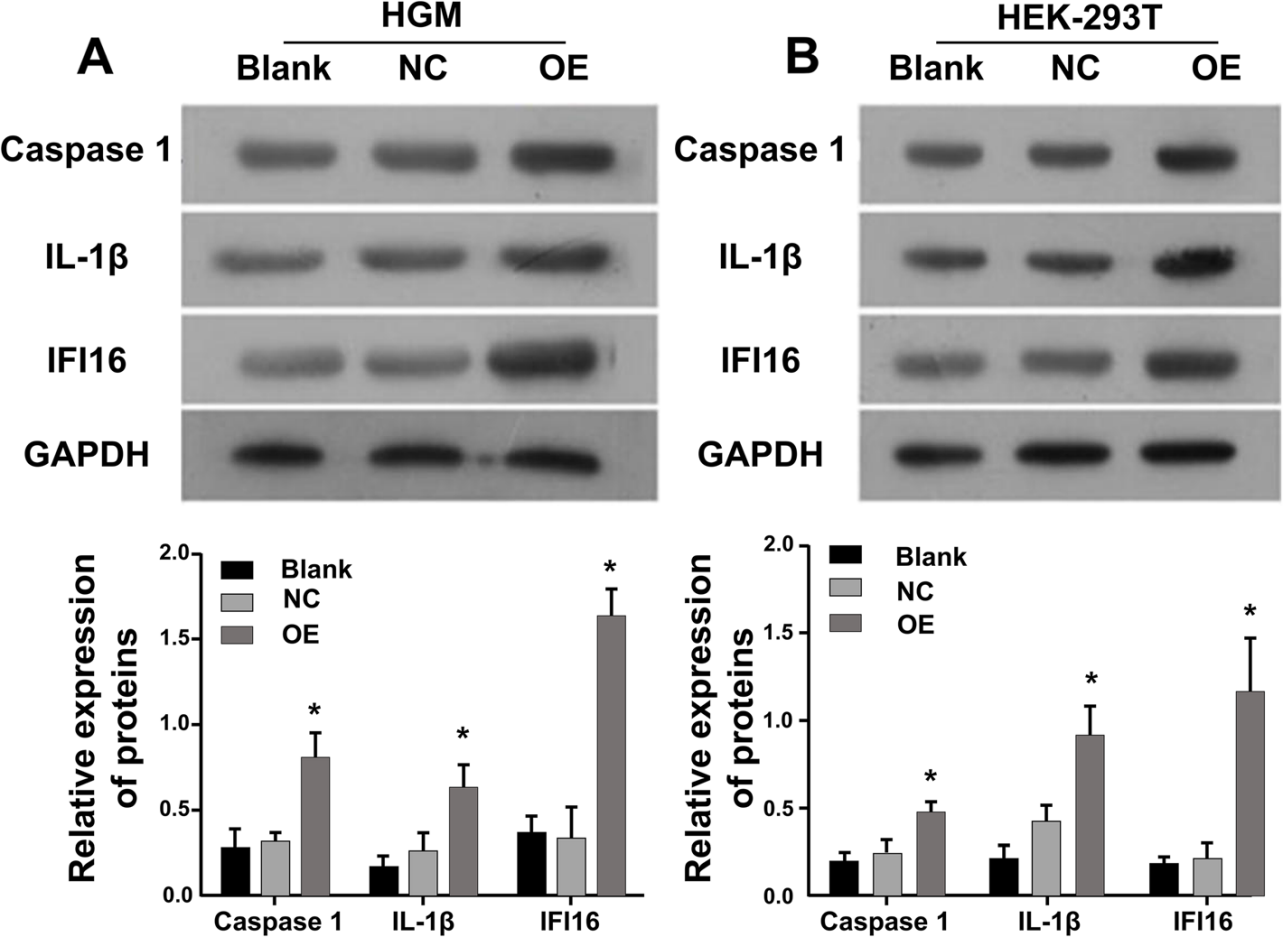
The expression of IFI16, Caspase-1 and IL-1ß protein levels significantly elevated in OE group compared those in NC and Blank group of the two cell lines, respectively

According to the components and structure of glomerular prophase cells, HGM cells and HEK-293 T cell lines were used to establish HBV infection model in vitro. The cells were divided into three groups: IFI16 and HBV DNA co-transfected group (over expression group, OE), HBVDNA-transfected group without IFI16 (negative control group, NC), and empty plasmids transfected group (blank group, Blank). The expression of IFI16, caspase-1 and IL-1 β were detected by Western blot. Results show that the expression of IFI16, Caspase-1 and IL-1ß protein levels significantly elevated in OE group compared those in NC and Blank group, respectively (Fig. 3A, B). The data indicated that over expression of IFI16 resulted in upregulation of Caspase-1 and IL-1ß protein levels.

### Knockdown of IFI16 reduced inflammation in vitro

**Fig. 4:**
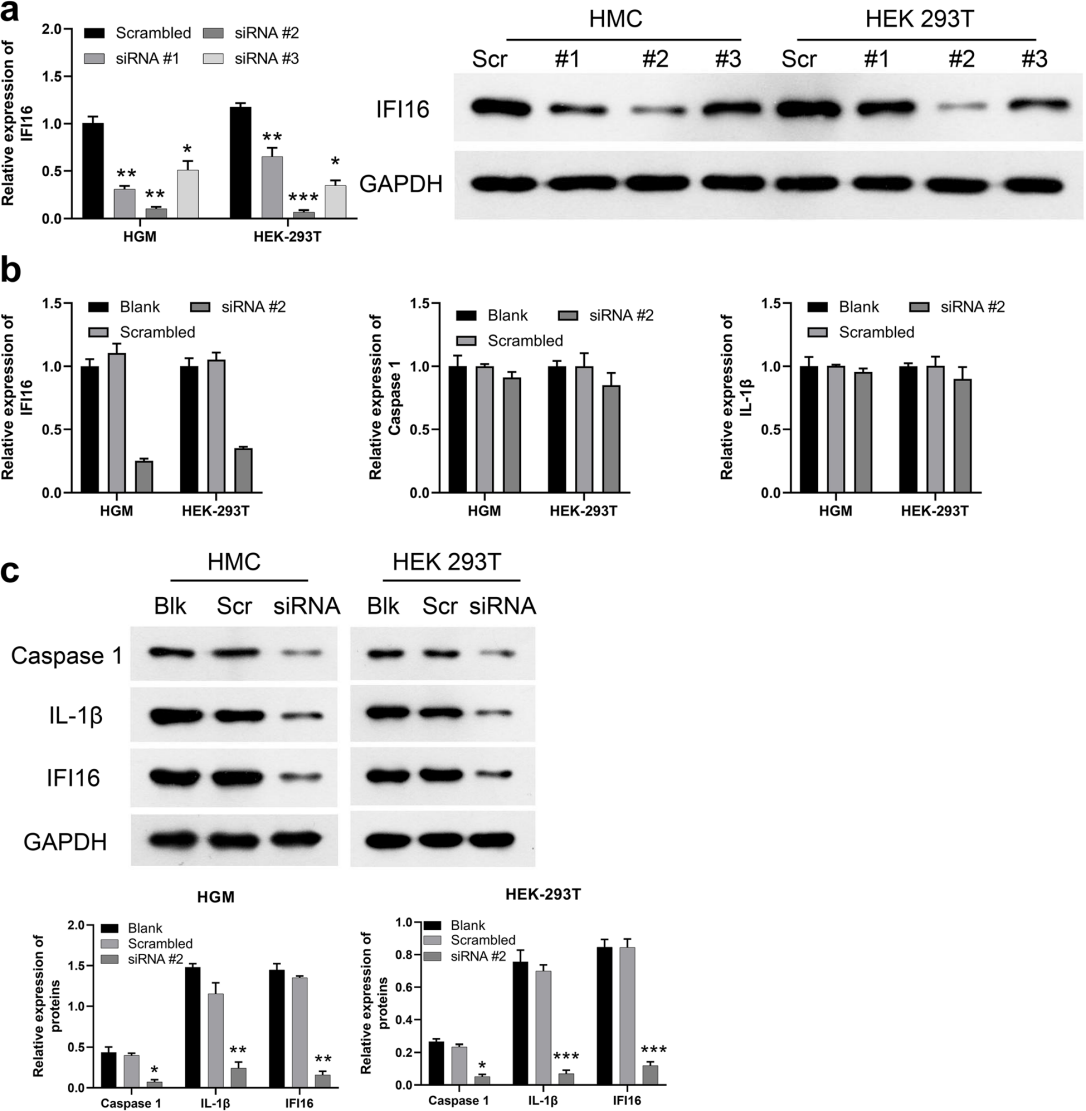
Down-regulation of IFI16 reduced the expression of caspase-1 and IL-1ß in siIFI16 group compared those in NC and Blank group of the two cell lines, respectively

To determine whether reduction in the IFI16 level could reverse the inflammation observed in the overexpression assay described above, we down-regulated the gene expression of IFI16 infected HGM cells and HEK-293 T cells using siRNA (siIFI16). the cell lines were divided into three groups: IFI16-siRNA and HBV DNA co-transfected group (si IFI16 group, si IFI16), HBVDNA-transfected group without siIFI16 (negative control group, NC), and empty plasmids transfected group (blank group, Blank). By 48 h post transfection, there was approximately 90% decrease of IFI16 mRNA levels in HGM cells and 95% decrease in HEK-293 T cells in the siIFI16 group (Fig. 4A). There is no reduction in caspase-1and IL-1훽 mRNA levels (Fig. 4B). Compared to blank group, siRNA-mediated knockdown of IFI16 resulted in a 6.14-fold decrease of caspase-1, a 6.16-fold decrease of IL-1훽 in HGM cells, and a 5.21-fold decrease of caspase-1, a 10.92-fold decrease of IL-1훽 in HEK-293 T cells (Fig. 4C).

## Discussion

Hepatitis B virus associated glomerulonephritis (HBV-GN) is one of the most common HBV infection associated diseases. it is present in 3–5% of patients with chronic HBV infection [17]. HBV-GN has been described and classified into four different subtypes, including membranous nephropathy, membranoproliferative glomerulonephritis and mesangial proliferative glomerulonephritis. However, the specific pathogenesis and role of HBV in HBV-GN is not yet clear. HBV contains a circular and partially double-stranded DNA (dsDNA). Increasing immunological evidence and electron microscope findings have suggested that HBV-DNA replication was detected directly in HBV-GN kidney tissue [18, 19]. HBV is not directly cytopathic in HBV-GN. The widely accepted view is that immune complex including circulating and in situ immune complexes depositing in basement membrane and/or in the mesangium induces immune damage [20]. and the autoimmune response may play an important role in the pathogenesis of HBV-GN [21, 22]. More recently, several reports have indicated that HIN 200 family (hematopoietic interferon-inducible nuclear proteins with a 200-amino acid repeat) appear to be involved in this autoimmunity [23].

HIN 200 family is defined by one or two C-terminal DNA binding HIN domains and an N-terminal homotypic protein–protein interactions pyrin domain (PYD). Currently, HIN 200 family includes four members: AIM-2 (absent in melanoma 2), IFIX (interferon-inducible protein X), MNDA (Myeloid cell nuclear differentiation antigen) and IFI16.Previous studies showed that AIM2 can stimulate inflammasome activation and interact with ASC [24]. the AIM2-ASC inflammatory signaling pathways activation may occur during HBV infection [25]. Notably, AIM2 has been shown to be a receptor for HBV DNA that regulates activation of caspase-1,which leads to the release of IFN- β [13]. our previous work also showed the expression of AIM2 was significantly higher in HBV-GN patient kidney tissues, the binding of HBV DNA to AIM2 appears to trigger immune response causing renal damage [23, 26]. IFI16 is related to the DNA sensor AIM2, and its surface is similar to AIM2 to bind dsDNA [27]. What’s more, IFI16 is the only member of this family with two HIN domains, which potentially increase the DNA-binding ability [28] and the immune damage.

In this study we described that the expression of IFI16 significantly elevated in HBV-GN kidney tissues compared with that in CCN kidney tissues. Interestingly, we found the IFI16 expression was not significantly different between the HBV parameters or the HBV-GN subtypes, The possibility is that beyond activation of IFI16, HBV DNA may have other actions. Just like human cytomegalovirus (HCMV) has positive and negative effects on IFI 16 for HCVM tegument protein pUL83 can disrupt IFI16 and inhibit the immune reaction [29].

Further we found IFI16 was localized exclusively in the nucleus. This was not similar to SLE which IFI16 was localized in the cytoplasm of keratinocytes [30], IFI16 expression in normal skin was restricted to the nuclei, while translocated to the cytoplasm in SLE and ultraviolet-induced cell injury. Furthermore, during human cytomegalovirus (HCMV) infection, IFI16 translocated into the cytoplasm during early stage and improved vesicle sorting and binding ability [31]. The overexpression and extranuclear appearance of IFI16 leads to the formation of inflammasomes and eventually induced specific autoimmune reaction cascade [30]. Thus this translocation of IFI16 is likely to enhance the protein function.

Whether HBeAg status in serum influencing IFI16 activation and binding to HBV-DNA was evaluated, Our results showed that the expression of IFI16 was not significantly different between the HBeAg positive and negative groups, demonstrating that IFI16 activation and binding to HBVDNA is not influenced by serum HBeAg status. Moreover, our results indicated that the positive expression rate of IFI16 showed no difference between in the high replication group (HBV-DNA ≥ 1 × 105 copies/ml) and the low replication group (HBV-DNA < 1 × 105 copies/ml), suggesting that high HBV load in serum does not mean a high load in the kidney tissue, on the other hand, it may be that HBV only plays a role of initiator, and does not mean that a high viral load will lead to the release of more severe inflammatory factors. We also considered another potential influence factor, whether the expression of IFI16 is different in different pathological types, the results showed IFI16 expression was negatively correlated with pathological types. Mesangioproliferative glomerulonephritis (MsPGN), Membranoproliferative glomerulonephritis (MPGN), Membranous nephropathy (MN) and Minimal change glomerulopathy.(MCG) were distributed in HBVGN pathological diagnosis. In this study, the pathological types of hepatitis B associated kidney were mainly membrane hyperplasia; its inflammation is not only shown in the glomerulus, but also in the renal interstitium, where the inflammatory cell infiltration is more, from focal to multi-focal. IFI16 expression showed was negatively correlated with pathological types.

We found that the expression of IFI16 was positively correlated with expression of Caspase-1and IL-1ß, which may then be responsible for the renal damage seen in HBV-GN patients. We infer that after sensing dsDNA, IFI16 leads to the formation of inflammasomes [7], which also causes the activation of caspase-1 and subsequently the maturation and secretion of IL-1ß. IL-1ß is not only crucial to innate immune defense, but also an important mediator of adaptive immune response to viral infection [32]. Thus Caspase-1/IL-1ß pathway is critical for the clearance of pathogens or damaged cells in HBV-GN, and IFI16 may play an important role in disease pathogenesis.

To further verified if the IFI16 induces the ASC-dependent inflammasome pathway through the Caspase-1/IL-1ß pathway leading kidney injure, first we analyzed the expression of IFI16, Caspase-1and IL-1ß in over expression IFI16 cells transfected with HBV, the results demonstrated that protein levels of IFI16, Caspase-1and IL-1ß significantly elevated in OE group compared those in NC and Blank group, respectively. But the mRNA levels of Caspase-1and IL-1ß remained unchanged. Then we used siRNA-mediated knockdown of IFI16 cells transfected with HBV, we see that the downregulation of IFI16 in the two cell lines could directly inhibit the expression of caspase-1 and IL-1ß at protein levels, not mRNA levels. The results suggested that this inflammation signal transfer pathway was related to IFI16 levels in HBV-GN, and the intrinsic mechanism of Caspase-1/ IL-1ß activation is not in transcription level but the self-cleavage induced by IFI16. However, the specific interactions that occur between IFI16 and Caspase-1/ IL-1ß activation in HBV-GN are still unknown and need further exploration.

## Conclusions

In summary, our study revealed the expression of IFI16 significantly increased in HBV-GN patients. The elevation of IFI16 during HBV infection or replication may contribute to renal damage by regulating the Caspase-1/IL-1β pathway, which induces the inflammatory response. Our findings suggest that IFI16 plays an important role in the pathogenesis of HBV-GN, which may serve as a target molecule for diagnosing and treating for HBV-GN.

## Data Availability

The data sets used and/or analyzed during the current study are available from the corresponding author on reasonable request.

## Abbreviations

HBV: Hepatitis B virus
HBV-GN: Hepatitis B virus associated glomerulonephritis
CN: Chronic nephritis
CCN: Chronic glomerulonephritis
PHI-200: Pyrin-Hin200 family
IFN-I: Type I interferon
IFI16: Interferon-g-inducible protein 16
ASC: Apoptosis-associated speck-like protein
IL-1β: Interleukin-1β
HGM: Human glomerular mesangial
VACV: Vaccinia virus
HSV-1: Herpes simplex virus 1
KSHV: Kaposi sarcoma-associated herpesvirus
dsDNA: Double-stranded DNA
HAV: Hepatitis A virus
HCV: Hepatitis C virus
HEV: Hepatitis E virus
HIV: Human immunodeficiency virus;HSV:Herpes simplex virus
HCMV: Human cytomegalovirus
K/DOQI: Kidney Disease outcome quality initiative
NKF: National Kidney Foundation
H&E: Hematoxylin and eosin
PASM: Periodic acid-sliver methenamine
MsPGN: Mesangio proliferative glomerulonephritis
MPGN: Membranoproliferative glomerulonephritis
MN: Membranous nephropathy
MCG: Minimal change glomerulopathy
FSS: Focal segmental sclerosis
